# The Safety, Immunogenicity, and Immunopersistence of Hepatitis A Vaccine in HBs-Ag-Positive Participants: A Retrospective Study

**DOI:** 10.3389/fcimb.2021.672221

**Published:** 2021-06-17

**Authors:** Xiaodan Wang, Jia Luo, Fubao Ma, Guodong Kang, Zhengrong Ding, Yue Pan, Yujiao Zhao, Junying Chen, Kai Feng, Lingmei Yan, Juan Zhang, Linhao Li, Qiangping Lan, Daiying Li, Xiaolei Yang, Guoliang Li, Jingsi Yang, Qiangming Sun

**Affiliations:** ^1^ Institute of Medical Biology, Chinese Academy of Medical Sciences, and Peking Union Medical College, Kunming, China; ^2^ Yunnan Key Laboratory of Vaccine Research and Development on Severe Infectious Diseases, Kunming, China; ^3^ Yunnan Provincial Key Laboratory of Vector-borne Diseases Control and Research, Kunming, China; ^4^ Vaccines and Immunization Department, Jiangsu Provincial Center of Disease Control and Prevention, Nanjing, China; ^5^ Vaccines and Immunization Department, Yunnan Provincial Center of Disease Control and Prevention, Kunming, China; ^6^ School of Basic Medicine, Kunming Medical University, Kunming, China

**Keywords:** hepatitis A vaccines, immunogenicity, immune persistence, hepatitis B surface antigen, safety

## Abstract

**Objectives:**

To compare the safety, immunogenicity, and immune persistence of hepatitis A (HA) vaccines between HBs-Ag-positive and -negative participants.

**Method:**

9000 participants were enrolled in the phase IV study of live attenuated HA (HA-L) or inactivated HA (HA-I) vaccines. The HBs-Ag-positive subjects were detected and became an independent observation group. Adverse reactions (ARs), geometric mean concentrations (GMCs) and seroconversion rates (SRs) of the vaccines were analyzed at five time points until three years after vaccination. Results: 120 HBs-Ag-positive subjects were screened out, only 1 participant had grade 1 experienced ARs after HA-L injection. Except the time point of two years, the SRs of HBs-Ag-positive group were 100% for both vaccines. The GMCs were not statistically different between HBs-Ag-positive and -negative groups after the HA-L vaccination. The logarithmically transformed GMCs for HBs-Ag-positive and -negative groups were 3.21 mIU/mL (95% CI, 2.03-4.39 mIU/mL) and 2.95 mIU/mL (95% CI, 2.88-3.02 mIU/mL) 28 days after the HA-L vaccination, respectively.

**Conclusions:**

Both HA-L and HA-I vaccines were safe for HBs-Ag-positive participants and may provide an excellent long-term protection against HAV in this study. The results indicated that people positive or negative for HBs-Ag can receive both HA-L and HA-I vaccines (ClinicalTrials.gov number, NCT02601040).

## Introduction

China is a high epidemic region of viral hepatitis. Each year, approximately 1.5 million people are infected by hepatitis A (HA) virus (HAV). Acute HAV infection usually presents itself as a self-limiting infection with fatality rates varying from 0.01% to 0.5% in adults (da Silva et al., 2016). According to the WHO, HAV has resulted in 7134 deaths in 2016 (WHO, 2020a). HA is a feco-orally transmitted disease related to inadequate hygienic and sanitary conditions (Chen et al., 2017). With the development of economy, these conditions have already been improved, and HAV infections have been enormously reduced in China. However, HA vaccine continues to play a substantial role in reducing the infection because vaccination develops immunity to combat a sudden HAV outburst. Both live attenuated HA (HA-L) and inactivated HA (HA-I) vaccines used in this study were approved by the China Food and Drug Administration (CFDA). The HA vaccines were integrated into the Chinese national expanded immunization programme and were made freely available to all children above 18 months of age in 2008 (Cui et al., 2014; Ma et al., 2016).

Hepatitis B (HB) virus (HBV) causes a significant public health problem and has been widely distributed around the world. The global prevalence of HB surface antigen (HBs-Ag) ranges from 2% to 20%, and approximately 257 million people are living with an HBV infection (defined as HBs-Ag-positive) (Nguyen et al., 2020; Tout et al., 2020). HBV is transmitted through contact with blood or other body fluids of an infected person (WHO, 2020b). Although HBV vaccine was integrated into the Chinese national expanded immunization programme in 2002 (Liang et al., 2013), there continues to be a large number of HBs-Ag-positive people in China. The prevalence of HBs-Ag is around 0.5% in subjects ≤ 10 years of age, but the total prevalence of HBs-Ag has been stabilized at approximately 6.0% in Beijing from 2008 to 2018 (Xu et al., 2020). Even the combined vaccine of HAV and HBV has been on the market, there was still lack of clinical date for the HAV safety and effectiveness in HBs-Ag positive people. When the HBs-Ag positive participants received HA vaccines, the patients always consulted about the safety and effectiveness of hepatitis A vaccine in HBs-Ag positive. One of the aims of this study was to evaluate whether these adverse reactions (ARs) were exacerbated by previous hepatitis B infection. In recent years, it has been found that HAV can significantly inhibit HBV replication when HBV infected patients are co-infected with HAV. Former studies shown that HAV vaccine has a certain clinical therapeutic effect on HBV positive patients (Hang et al., 2000). But could the HBs-Ag positive affect the effectiveness of HAV vaccine? It has been reported that previous hepatitis B infection and the persistence of hepatitis B antigen may activate the immune system (Liaw and Chu, 2009). Some studies have suggested that all patients with HBV and HCV infections should also be specifically considered for vaccination against HAV (Department of Health and Human Services, Centers for Disease Control and Prevention and Advisory Committee on Immunization Practices (ACIP); Advisory Committee on Immunization Practices (ACIP) et al., 2006; Moorman et al., 2018). Could HBV-induced immune activation impact immunogenicity and immunopersistence of HA vaccines?

Previous studies have investigated the safety, immunogenicity and immunopersistence of HA vaccines (Shah et al., 2020; Wang et al., 2020). We have previously compared the safety, immunogenicity, and persistence between HA-I and HA-L vaccines (Ma et al., 2016; Luo et al., 2019), however, the enrolled participants did not exclude the HBs-Ag-positive population. We suggest that the comparison of safety, immunogenicity, and immune persistence between HBs-Ag-positive and -negative participants is meaningful. Those comparison can provide some useful insights with respect to the use of HA vaccines.

## Methods

### Ethics Statement

Institutional Review Board approval was obtained from the Ethics Committee of the Jiangsu Provincial CDC, China. All enrolled participants provided written informed consent before commencing with any study procedures. This trial is registered with Clinical Trials. gov, number NCT02601040.

### Exclusion Criteria

The exclusion criteria included participants with known infectious diseases (excluding HBs-Ag positive), serious chronic illnesses or developmental disorders, nervous system damage, congenital malformation, and thrombocytopenia or other coagulation disorders. Participants were also excluded if immunoglobulin or any research vaccine or drug was administered during the clinical trial.

### Vaccines

Both HA-L and HA-I vaccines were manufactured by the Institute of Medical Biology, Chinese Academy of Medical Sciences, China. The vaccines were prepared from the Lu8 and H2 strains, respectively. The vaccines were cultured in the human diploid cell line (KMB17 strain) for proliferation. There were two specifications for the HA-I vaccine; one, contained 320 ELISA units (EU) of inactivated HA viral antigen adsorbed to 0.5 mg of aluminum hydroxide and suspended in 0.5 mL of buffered saline for babies and children aged 18 months to 16 years, the other, contained 640 EU for adults aged 16 years or older. The HA-L vaccine contained 6.50 lgCCID50/mL of the HA viral antigen adsorbed to 0.5 mg of aluminum.

### Study Design and Participants

**Figure 1:**
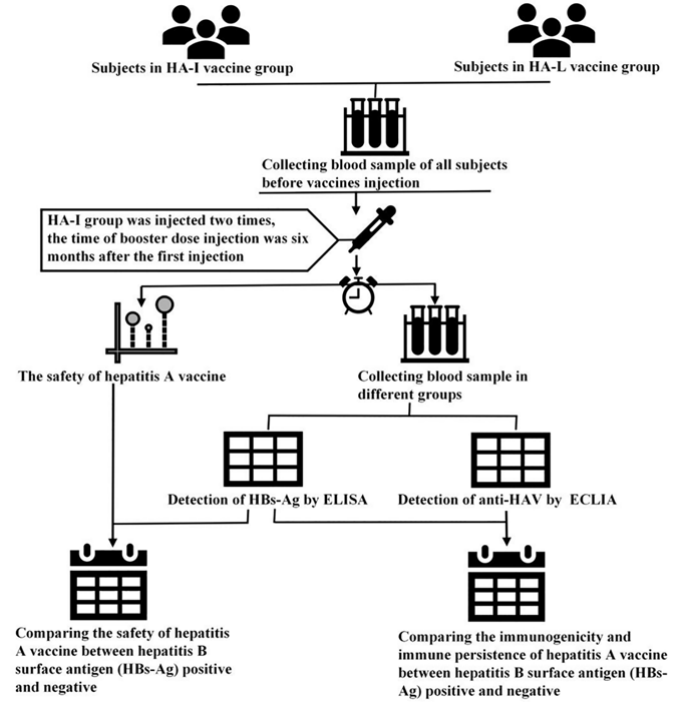
The research strategy of this study.

This double-blinded, cluster-randomized clinical trial was conducted in the Jiangsu Province in China. All enrolled participants and investigators did not know which vaccines were injected at the first dose of the vaccine. The staffs involved in this experiment were professionally trained. A total of 9000 participants were enrolled for this study, and there were 4500 participants each group (17). To evaluate the safety and immunopersistence of the HA-I and HA-L vaccines in HBs-Ag positive and -negative participants, 8763 participants were selected for the safety observation after vaccination and 1800 were randomly selected for serum detection (**Figure 1**). Thus, serum samples were collected for anti-HAV IgG and HBs-Ag detection at the prescribed times (0 days (before vaccination), 28 days, 1 year, 2 years, 3 years).

### Immunogenicity and Immunopersistence of HA Vaccines

Immunogenicity and immunopersistence analyses were performed on participants in a randomly selected subgroup for each type of HA vaccine. Serum samples were collected at the prescribed time points and levels of the HA IgG antibody (anti-HAV IgG) were measured by electrochemiluminescence immunoassay (ECLIA; Roche Diagnostics GmbH). Participants with anti-HAV IgG levels greater than or equal to 20 mIU/mL was defined as seroconversion.

### The Detection of HBs-Ag

Serum HBs-Ag was detected using an enzyme-linked immunosorbent assay (ELISA, Kehua Bio-Engineering company, Shanghai, China). According to the instruction of ELISA, the HBs-Ag positive was estimated as per the instruction.

### Safety Assessment

Participants who received an HA-L or HA-I vaccination were monitored for immediate ARs for at least 30 min by the local Centers for Disease Control and Prevention (CDC). The ARs were monitored from day 1 (date of vaccination) to day 28. Participants were instructed to take daily axillary temperature measurements and asked to report any ARs or suspected unexpected serious ARs to the local CDC staff, who were asked to record any reported ARs in the Vaccination Report Booklet within 28 days after each vaccine dose. The categories of ARs have been shown in **Table 1**. The ARs were coded and graded according to the guidelines provided by the CFDA and the Division of Microbiology and Infectious Diseases of the National Institutes of Allergy and Infectious Diseases (Notice the Standard Guidelines or Adverse Reactions Grading of Vaccine Clinical Trials; National Institute of Allergy and Infectious Diseases, 2007).

**Table 1:** The categories of ARs (adverse reactions) after vaccination.

ARs	The categories of ARs			
	grade 1	grade 2	grade 3	grade 4
Induration/erythema/swelling/rash	<10 mm	10-25 mm	26 -50 mm	>50 mm
Fever	37.1°C to <37.6°C	37.6°C to <39.0°C	39.0°C>	
Pain and pruritus at the injection site, skin and mucosa, nausea, allergy, vomiting, diarrhea, daily activities, cognitive reactions, breastfeeding disorders	Symptoms were easily tolerated and did not interfere with daily activities	Sufficient discomfort to cause some interference with daily activities	Incapacitating, with inability to work or perform usual activities	Potentially life-threatening syndrome requiring emergency treatment or hospitalization

### Statistical Analysis

For the safety analysis, ARs were reported in units of each subject, and calculated as the number of subjects rather than ARs; that is if the same event occurred repeatedly in a subject during a follow-up, the event was counted only once. The ARs that occurred repeatedly in the same subject were summarized according to the following principles: if a subject repeated the same ARs more than once, the most serious degree and the closest relationship with the vaccination was taken. Hereby, AR rate (%) = number of responders with at least 1 occurrence/the number of randomized and vaccinated patients in the group, and incidence of ARs (%) = number of symptoms of an AR/the number of randomized and vaccinated patients in the group were used. The ARs were compared using continuous calibration Chi-squared detection.

For immunogenicity and immunopersistence analyses, participants were included if they completed the entire course of immunization and underwent a serum sample collection pre- and post-vaccination in accordance with the study design. An antibody titre of 20 mIU/mL was proposed in this study as the threshold for protection from HAV infection, based on other studies (Luo et al., 2019). The seroconversion rates (SRs) and geometric mean concentrations (GMCs) of anti-HAV antibodies were calculated. The SRs were defined as the number of participants who were initially anti-HAV seronegative and whose anti-HAV titres increased to 20 mIU/mL or more after administration of the vaccine. The Seroconversion rates (SRs) were compared by the Fisher’s exact test, while geometric mean concentrations (GMCs) were compared by the Student’s t-test after the anti-HAV titers had been logarithmically transformed.

The significance level for p values was 0.05 (two-sided). All data analyses were performed using the SPSS version 20.0 (IBM, Armonk, New York, The United States) and GraphPad Prism 7. A P value <0.05 was considered significant.

## Results

### Detection of HBs-Ag

**Figure 2:**
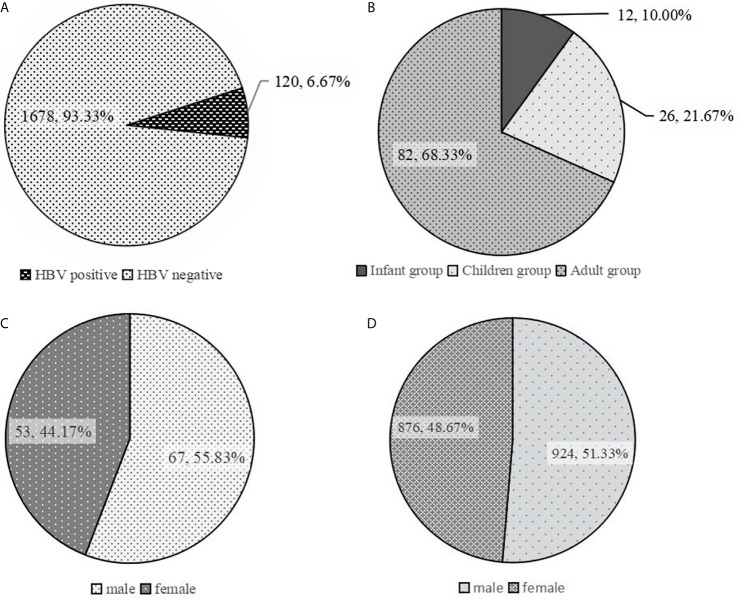
Analysis of characteristics of HBs-Ag-positive participants. **(A)** The proportion of HBs-Ag-positive participants in all participants to be tested. **(B)** The age distribution in HBs-Ag-positive participants. **(C)** The proportion of males and females in HBs-Ag-positive participants. **(D)** The proportion of males and females in all participants to be tested.

The results from ELISA detection revealed that 120 out of the 1800 participants were HBs-Ag positive. There were 69 and 51 HBs-Ag-positive participants in the HA-L and HA-I groups, respectively. The rate of HBs-Ag-positive participants was 6.67% (120/1800) at first dose of vaccine. Of the 120 HBs-Ag-positive participants, there were 82, 26, and 12 participants in the adult, children, and infant groups, respectively (**Figures 2A, B**). The total number of participants in different age groups was the same (600 per group), and the proportion of HBs-Ag-positive participants in the adult group was much higher than that in the children and infant groups. Moreover, the proportion of males in the gender composition ratio was 55.83% in 120 HBs-Ag-positive participants, which was higher than 51.33% in all 1800 participants (**Figures 2C, D**). There was no statistically significant difference in gender composition between the HBs-Ag-positive participants and all testing participants (P>0.05).

### Safety

Solicited injection site ARs included pruritus, pain, skin and mucosa induration, erythema, swelling, and rash. The solicited systemic ARs included fever, allergy, mental reactions, vomiting, daily activities affected, convulsion, unusual crying, diarrhea, nausea, and breastfeeding disorders. Of the 8763 participants from both HA-I (two dose) and HA-L groups, 381 experienced grades 1 or 2 ARs; only a one-year-old boy had grade 3 AR with a fever and body temperature of 39°C. The comparison of ARs between groups HA-L and HA-I (first dose), HA-L and HA-I (second dose), and HA-I (first dose) and HA-I (second dose) indicated that there were no statistically significant differences (P>0.05). Among the ARs, there were 4 in HBs-Ag-positive participants, of which 3 were in the HA-L group and 1 was in the HA-I group, as shown in **Table 2**. In the HA-L group, the AR in HBs-Ag-positive participants was 4.35% (3/69), which was higher than that of the participants in the HA-I group [1.96% (1/51)]. In the HA-L group, the AR in HBs-Ag-positive participants was 4.35% (3/69), which was higher than that of the HBs-Ag-negative participants [3.05% (135/4431)]. In the HA-I group, the AR in HBs-Ag-positive and HBs-Ag-negative participants was 1.96%(1/51) and 2.63% (117/4449), respectively.

**Table 2:** The comparison of adverse reactions (ARs) in HBs-Ag-positive or -negative participants between different groups.

HBs-Ag	no. of participants with ARs (%)			χ^2^ value	P value
	HA-L	HA-I (first dose)	HA-I (second dose)		
Positive	4.35% (3/69)	1.96% (1/51)	0	0.042^a^ -^b^ -^c^	P>0.05 - -
Negative	3.05% (135/4431)	2.63% (117/4449)	2.98% (125/4212)	1.399^a^ 0.046^b^ 0.910^c^	P>0.05
Total	3.10% (138/4500)	2.62% (118/4500)	2.93% (125/4263)	1.387^a^ 0.136^b^ 0.780^c^	P>0.05

The comparison of ARs between HA-L and HA-I (first dose) (a), HA-L and HA-I (second dose) (b), and HA-I (first dose) and HA-I (second dose) (c), were conducted by a continuous calibration Chi-squared detection.

### Immunogenicity and Immunopersistence of HA Vaccine in HBs-Ag-Positive Participants

After excluding participants with anti-HAV IgG levels higher than 20 mIU/mL before vaccination, there were 16 HBs-Ag-positive participants that were included for the assessment of immunogenicity and immunopersistence. The anti-HAV IgG SRs and GMCs were calculated at 28 days, 1 year, 2 years, and 3 years after vaccination; booster injections containing HA-I vaccines were administered again at 6 months.

Among the HBs-Ag-positive participants in the HA-L group, the SRs reached 100.00% at 28 d, 1 year, and 3 years following immunization, while at 2 years the SRs were 80.00% (95% CI: 24.47-135.53) (**Table 3**). Among the HBs-Ag-positive participants in the HA-I group, the SRs reached 100.00% at 28 d after the first dose and the booster injection and remained 100.00% at 1 year, 2 years, and 3 years following immunization. Among HBs-Ag-negative participants in the HA-L group, the SRs reached 98.06% (95% CI: 96.12-99.61) at 28 d and remained 99.62% (95% CI: 98.49-99.62) at 1 year, 97.39% (95% CI: 95.15-99.25) at 2 years, and 99.62% (95% CI: 98.46-99.62) at 3 years following immunization. Among the HBs-Ag-negative participants in the HA-I group, the SRs reached 99.63% (95% CI: 98.53-99.63) 28 d after the first dose, 100.00% at 28 d after the booster injection and 1 year, 96.63% (95% CI: 94.57-98.70) at 2 years, and 100% at 3 years following immunization (**Table 3**).

**Table 3:** The immunogenicity and immunopersistence of HA vaccines in HBs-Ag-positive participants.

Vaccine groups	Sampling time point	HBs-Ag-negative	HBs-Ag-positive	p-value
HA-L	Before vaccination			
	SRs (seroconversed/detected)	_	_	NS
	SR % (95% CI)	_	_	
	GMCs, mIU/mL (95% CI)	0.81 (0.78-0.84)	0.79 (0.56-1.01)	
	28 days after vaccination			
	SRs (seroconversed/detected)	253/258	3/3	
	SR % (95% CI)	98.06 (96.12-99.61)	100	NS
	GMCs, mIU/mL (95% CI)	2.95 (2.88-3.02)	3.21 (2.03-4.39)	
	1 year after vaccination			
	SRs (seroconversed/detected)	264/265	5/5	NS
	SR % (95% CI)	99.62 (98.49-99.62)	100	
	GMCs, mIU/mL (95% CI)	3.14 (3.06-3.22)	3.04 (2.44-3.63)	
	2 years after vaccination			
	SRs (seroconversed/detected)	261/268	4/5	
	SR % (95% CI)	97.39 (95.15-99.25)	80.00 (24.47-135.53)	NS
	GMCs, mIU/mL (95% CI)	3.64 (3.55-3.73)	3.09 (1.33-4.85)	
	3 years after vaccination			
	SRs (seroconversed/detected)	259/260	5/5	
	SR % (95% CI)	99.62 (98.46-99.62)	100	NS
	GMCs, mIU/mL (95% CI)	3.69 (3.62-3.77)	3.67 (2.71-4.63)	
HA-I	Before vaccination			
	SRs (seroconversed/detected)	_	_	
	SR % (95% CI)	_	_	NS
	GMCs, mIU/mL (95% CI)	0.82 (0.79-0.85)	0.76 (0.58-0.94)	
	28 days after the first dose			
	SRs (seroconversed/detected)	272/273	4/4	
	SR % (95% CI)	99.63 (98.53-99.63)	100	NS
	GMCs, mIU/mL (95% CI)	2.96 (2.91-3.02)	3.04 (2.60-3.48)	
	28 days after the booster dose			
	SRs (seroconversed/detected)	309/309	4/4	
	SR % (95% CI)	100	100	NS
	GMCs, mIU/mL (95% CI)	3.39 (3.34-3.44)	3.39 (3.05-3.74)	
	1 year after the booster dose			
	SRs (seroconversed/detected)	309/309	4/4	
	SR % (95% CI)	100	100	NS
	GMCs, mIU/mL (95% CI)	3.58 (3.50-3.66)	3.59 (3.11-4.07)	
	2 years after the booster dose			
	SRs (seroconversed/detected)	287/297	6/6	
	SR % (95% CI)	96.63 (94.57-98.70)	100	NS
	GMCs, mIU/mL (95% CI)	3.53 (3.43-3.62)	3.50 (3.04-3.96)	
	3 years after the booster dose			
	SRs (seroconversed/detected)	309/309	4/4	
	SR % (95% CI),	100	100	NS
	GMCs, mIU/mL (95% CI)	3.87 (3.82-3.91)	3.99 (3.87-4.11)	

a. Seroconversion rates (SRs) were compared by the Fisher’s exact test, while geometric mean concentrations (GMCs) were compared by the Student’s t-test after the anti-HAV titers had been logarithmically transformed.

b. No statistically significant differences, p > 0.05.

**Figure 3:**
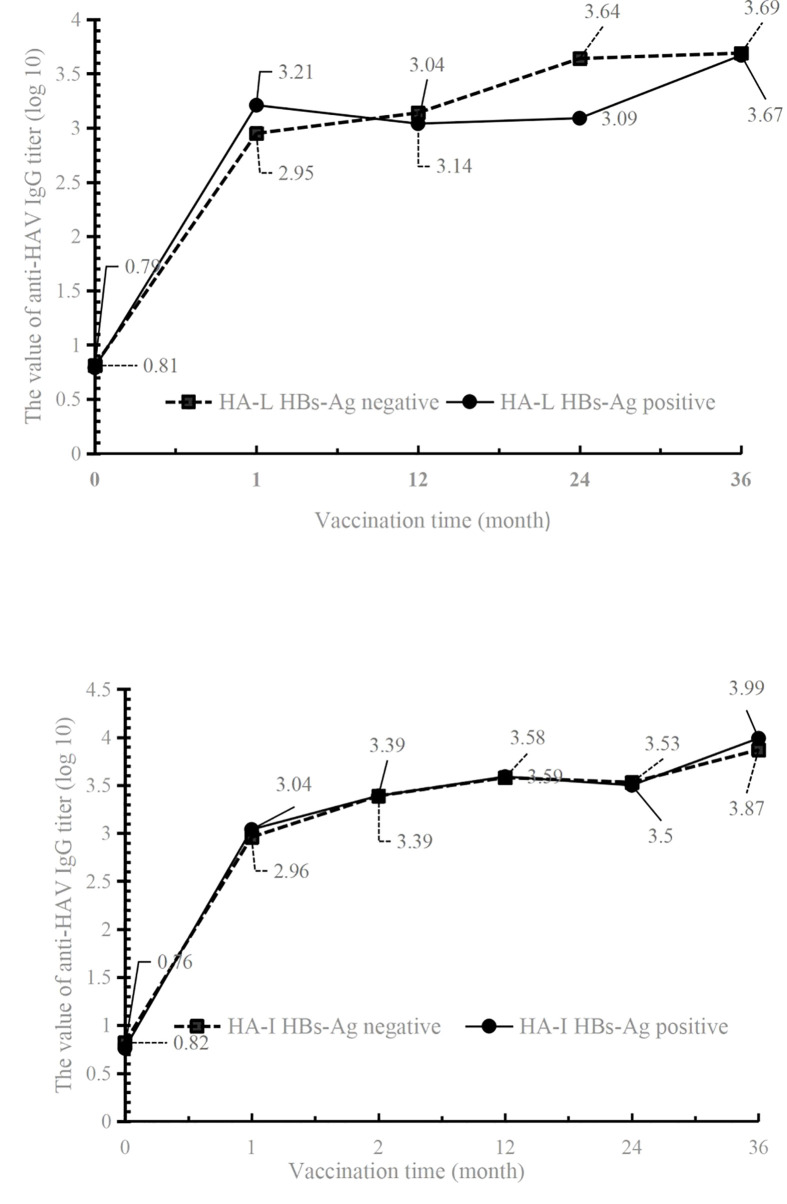
The values of anti-HAV IgG titer in HBs-Ag-positive or -negative participants of HA-L and HA-I groups.

The results showed that in the HBs-Ag-positive or -negative populations of both HA-L and HA-I groups, the anti-HAV IgG titer was drastically increased. Three years after inoculation, high levels of antibody titers were maintained in both groups, indicating that vaccines could provide good protection (**Figure 3** and **Table 3**). The antibody titer did not decrease significantly at the time points 1 year, 2 years, and 3 years after immunization, therefore, the effective protective time of antibody could not be obtained by a statistical analysis.

## Discussion

HAV is a linear single-stranded RNA and a member of the genus *Hepatovirus* in the family *Picornaviridae* (Jacobsen and Wiersma, 2010). HBV is a hepatotropic non-cytopathic DNA virus, and unlike a self-limiting HAV infection, HBV generates a covalently closed circular DNA (cccDNA) intermediate in the nucleus of infected cells and integrates sequences that act as transcription templates for viral proteins (Fanning et al., 2019). According to the WHO, most HBV-infected persons become chronic carriers; 27 million people (10.5% of all the people estimated to be living with HB) were aware of their infection (Fite et al., 2020; WHO, 2020b). Vaccination is the most effective approach of preventing infection with the HAV and HBV. As for China, both the HA (HA-I and HA-L) and HB vaccines were integrated into the Chinese national immunization program (NIP), provided for free to all children above 18 months of age (HA vaccine) or new-born babies (HB vaccine) (China Center for Disease Control and Prevention). The large-scale use of HB vaccine has dropped the number of HBs-Ag-positive individuals in the population, especially in infants and children. In our study, there were only 3.83% (26/600), 2% (12/600) and 13.67% (82/600) participants in the children, infant, and adult groups. These results indicated that the NIP was an effective approach to decrease the HBV infection.

Owing to the similarities between HA and HB in terms of their target organs and symptoms, whether the safety of HBV previous infected people would be compromised upon receiving HA vaccinations was our main concern. Since the HBs-Ag-positive population was only 120, which was smaller compared to the population of 1800 enrolled people, the huge disparity between HBs-Ag-positive and -negative led to no statistically significant differences. Moreover, all ARs in these 120 HBs-Ag-positive participants belong to grade 1 or 2, and followed good prognosis, no matter which vaccine groups they belonged to. The study was conducted in the Jiangsu province, and the locals prefer to eat shellfish and other seafood products. Several studies indicated that seafood products, especially shellfish, are easy to accumulate HAV. In 1988, a total of 38000 people were infected by HAV in Shanghai, which was caused by eating Scapharca subcrenata (WHO, 2012). This explained why there were greater numbers of participants whose anti-HAV IgG titers before vaccination was greater than 20 mIU/mL. Thus, according to our exclusion criteria, these participants were excluded in the subsequent analysis for immunogenicity and immunopersistence of HA vaccine. Consequently, the number of HBs-Ag-positive participants decreased markedly to 3 at one time point and therefore, could not compare the immunogenicity and immunopersistence between HBs-Ag-positive and -negative participants; however, the anti-HAV IgG titer in positive participants in both vaccine groups dramatically increased after 28 days of initial immunization and remained high at 1 year, 2 years, and 3 years following immunization. The results indicated that an HBs-Ag-positive status had no impact on the effectiveness of the vaccines.

Although the number of HBs-Ag-positive participants were only 120, the comparison of safety, immunogenicity, and immunopersistence led to no statistically significant difference between the HBs-Ag-positive and -negative participants. However, for those 120 participants, both HA vaccines were found to be safe with long-term immunogenicity and immunopersistence. This study demonstrated that an HBs-Ag-positive status may had no influence on the safety and the effectiveness of the vaccines. Thus, people positive or negative for HBs-Ag can receive both HA-L and HA-I vaccines. However, the number of participants for HBs-Ag positive is small, another study with more HBs-Ag-positive participants is warranted to support these conclusions in the future.

## Data Availability Statement

The original contributions presented in the study are included in the article/supplementary material. Further inquiries can be directed to the corresponding authors.

## Ethics Statement

The studies involving human participants were reviewed and approved by The Ethics Committee of the Jiangsu Provincial CDC, China. This trial is registered with Clinical Trials. gov, number NCT02601040. Written informed consent to participate in this study was provided by the participants’ legal guardian/next of kin.

## Author Contributions

QS and JY conceived and designed the study. XW, JL, FM, GK, ZD, YP, YZ, and JC performed the sampling and experiments. XW, JL, KF, LY, JZ, LL, QL, DL, XY, and GL analyzed the data. XW and QS prepared and reviewed the manuscript. All authors contributed to the article and approved the submitted version.

## Funding

This study was supported by the National “The 12th Five Year”Major New Drug Discovery’ Technology Major Projects (grant no.2012ZX09104-302), the Jointly Supported Foundation of the National Project in Yunnan Province (grant no. 2013GA018), the CAMS Initiative for Innovative Medicine (CAMS-I2M) (grant no. 2017-I2M-2-006), the National Natural Science Foundation of China (grant no. 31970868), the Major Science and Technology Projects of Yunnan Province (grant no. 2019ZF004), and the Youth Project in Yunnan Province (grant no. 2019FD082).

## Conflict of Interest

The authors declare that the research was conducted in the absence of any commercial or financial relationships that could be construed as a potential conflict of interest.
